# Epstein–Barr Virus in the Development of Colorectal Cancer (Review)

**DOI:** 10.17691/stm2021.13.4.09

**Published:** 2021-08-28

**Authors:** N.A. Oleynikova, N.V. Danilova, M.O. Grimuta, P.G. Malkov

**Affiliations:** Researcher, Department of Clinical Pathology; Medical Scientific and Educational Center, Lomonosov Moscow State University, 27/10 Lomonosov Prospect, Moscow, 119192, Russia; Senior Researcher, Department of Clinical Pathology; Medical Scientific and Educational Center, Lomonosov Moscow State University, 27/10 Lomonosov Prospect, Moscow, 119192, Russia; Student; I.M. Sechenov First Moscow State Medical University (Sechenov University), 8/2 Trubetskaya St., Moscow, 119991, Russia; Head of the Department of Clinical Pathology; Medical Scientific and Educational Center, Lomonosov Moscow State University, 27/10 Lomonosov Prospect, Moscow, 119192, Russia

**Keywords:** Epstein–Barr virus, colorectal cancer, the cycle of the virus, carcinogenesis, inflammatory diseases of the colon

## Abstract

The study of the influence of the Epstein–Barr virus (EBV) on the development of colorectal cancer is of current interest, particularly in light of the active discussion of the participation of this virus in the carcinogenesis of stomach cancer. In this review, aimed at a fundamental understanding of the processes associated with the impact of EBV on the human body, attention is paid to the issues of the life cycle of the virus, its phases (latent and lytic), as well as proteins that may be detected in each of the phases. The papers reporting on the role of EBV in the development of colorectal cancer have been analyzed. A summary table indicating the population under study, the number of samples, the method, and the result obtained is provided. Given that the primary cells affected by EBV are lymphocytes, it is logical to assume the involvement of this virus in the development of inflammatory bowel diseases. The review cites studies which confirm the presence of virus DNA in tissues in the inflammatory diseases of the colon, including microscopic and ulcerative colitis. To confirm the direct impact of EBV on the development of colorectal cancer, large studies with applying various methods for detecting the virus and the mandatory description of its localization are required. Besides, it is necessary to correlate these data with the clinical and morphological characteristics of EBV.

## Introduction

The Epstein–Barr virus (EBV) is the first detected and one of the most common oncoviruses which accounts for approximately 10–15% of all malignant neoplasms. It belongs to the group of gamma viruses and is ubiquitous in the adult population. The virus infects more than 90% of the world’s population by the age of 35, most being asymptomatic. The main pathway for its transmission is through the saliva [1].

The penetration of the virus into B lymphocytes located in the submucous lymphoid tissue (for example, in the tonsils) [2, 3] occurs when the EBV surface protein gp350 interacts with the CD21 receptor and HLA class II molecules. The way the virus “crosses” the mucous epithelial integument remains unclear, however, it has been established that, in addition to B cells, it is found in the epithelial cells of EBV-associated carcinomas, as well as in other hematopoietic cells (T cells, granulocytes, and natural killer cells) [4, 5].

The EBV causes infectious mononucleosis, some lymphoproliferative diseases (Burkitt’s lymphoma, Hodgkin’s lymphoma, T/NK cell lymphomas), as well as post-transplant lymphoproliferative conditions, promotes the development of epithelial neoplasms, including nasopharyngeal carcinomas and gastric carcinomas [6–8]. There are data on the expression of the virus in carcinomas of the breast, prostate, mouth, cervix, and salivary glands [9–14].

## EBV replication

The EBV genome consists of double-stranded DNA, which is approximately 172,000 bp in length [2]. It encodes viral oncogenes, such as EBV-encoded nuclear antigens (Epstein–Barr nuclear antigen, EBNA [15]) and latent membrane proteins (LMP) [16]. The main function of these proteins is to help maintaining genome replication by evading natural immune control mechanisms. Moreover, the corresponding *EBNA* and *LMP* genes have been found to play an important role as oncogenes in infected cells [2, 15, 17].

The EBV can replicate in two ways: by infecting B cells (latent form) and by lytic production of the virion (lytic form) [3, 18]. In its latent form, the viral DNA enclosed in a round plasmid behaves like the host chromosomal DNA and encodes viral genes, including six nuclear antigens (EBNA1, -2, -3A, -3B, -3C, and -LP), three latent membrane proteins (LMP1, -2A, and -2B), two small non-coding RNAs (EBER1 and -2), 44 miRNAs, and a BamHI-A transcript [19–21]. This leads to activation, proliferation of cells, and their resistance to “dying”. *In vitro* in B cells, the virus persists in a latent form with the expression of non-coding RNAs from viral DNA, thereby transforming B lymphocytes into “immortal” proliferating lymphoblastoid cell lines [22]. All the above-mentioned products have been found in lymphoblastoid cell lines, naive tonsillar B cells of healthy viral carriers, and almost all B cells of patients with infectious mononucleosis [3, 23–25].

**Figure 1:**
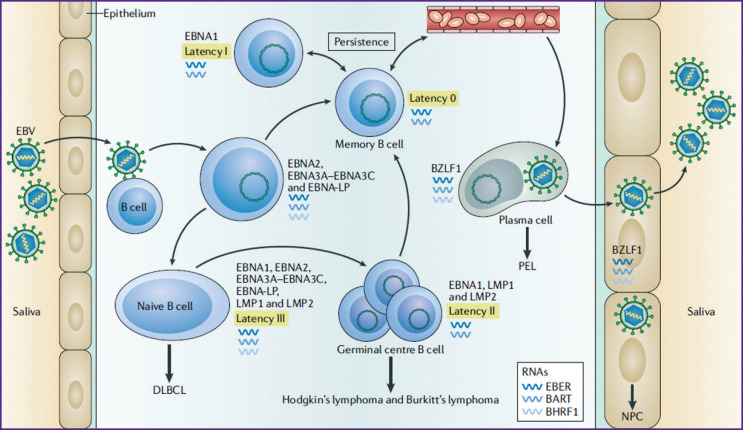
Model of latent infection of the Epstein–Barr virus. Given under [3] Here: PEL — primary effusion lymphoma

After crossing the mucous membrane of the epithelium, the salivary virus infects the B cells of the lymphoid tissue of the tonsils, which leads to EBNA2-dependent proliferation of the infected cells. Infected B cells can differentiate into latency 0 immediately after infection (EBV persists in circulating memory B cells without expressing viral particles). Transformation to latency III, during which EBNA1, EBNA2, EBNA3A–3C, EBNA-LP, LMP1, and LMP2 are expressed, is an alternative. After activation of latency III, B lymphocytes enter the germinal center of the lymphoid follicle. At this stage (called latency IIa), only three proteins (EBNA1, LMP1, and LMP2) are found in B lymphocytes. Latency  IIa can become latency 0, at which viral proteins are not produced. During cell division, which is in latency 0, latency I sets in, during which EBNA1 is present [26]. This phase is observed in healthy carriers of the virus and corresponds to a precancerous one [3]. Thus, four types of latent gene expression have been described for EBV, three of which (latency I, II, and III) are observed in malignant neoplasms associated with EBV [27]. According to some reports [4], latency I is most often associated with Burkitt’s lymphoma; latency II with Hodgkin’s lymphoma, T  cell non-Hodgkin’s lymphomas, and nasopharyngeal carcinoma (NPC), and latency III predominantly occurs in immunocompromised patients (e.g., post-transplant and AIDS-related lymphoproliferative disorders). According to the results of other studies [3], latency II is associated with diffuse large B cell lymphoma (DLBCL), and latency III — with Burkitt’s lymphoma and Hodgkin’s lymphoma (Figure 1).

In the lytic form, the EBV genome is sharply amplified (up to 1000 times) by the viral replication mechanism and selectively replicates the virion components (viral DNA — genomes and proteins). This process takes place in replicating compartments within the nucleus: the lytic program stops the cell cycle and significantly affects cellular processes. The lytic form of EBV infection is considered as a mechanism by which the virus induces neoplastic transformation into EBV-associated malignant neoplasms (carcinomas and lymphomas) [28]. This mechanism involves the transcription factors BZLF1 and BLIMP1, the latter of which is required for uncontrolled replication in epithelial cells (for example, in hairy leukoplakia of the oral cavity) [29]. In asymptomatic persistence of the virus, the lytic products of EBV, which subsequently stimulate the differentiation of B cells into plasma, are observed exclusively in plasma cells [3].

As already noted, the functions of proteins produced in the latent EBV form are different. Thus, EBNA1 is required for the initiation of viral genome replication during latent infection prior to the onset of mitosis. Then, this protein fixes the viral episome to the host chromatin during cell division in order to correctly distribute 10–40 viral particles from the mother B cell to the daughter one [30]. EBNA2 induces transcription of the cellular MYC oncogene and decreases lytic EBV replication through the induction of TET2 expression. EBNA-LP cooperates with EBNA2 to express viral oncogenes, including LMP1. EBNA3A and EBNA3C prevent the cell transition to lytic replication by suppressing BLIMP1 expression. On the contrary, EBNA3B provides sufficient tissue infiltration of immune cells around the EBV-transformed B cells to restrict the latter to a level where most asymptomatic EBV individuals do not develop lymphoma. LMP1 is a pro-proliferative and anti-apoptotic protein, and LMP2 provides a strong surface signal for B cell survival [3].

## EBV in the development of colorectal cancer

The similar structure and common histo- and embryogenesis of the stomach and other organs of the digestive tract suggest a possible role of EBV in the development of epithelial tumors, including colorectal cancer (CRC). The available literature contains less than 30 studies on the search for EBV in CRC samples (Table 1). Most of them were performed on small samples and have limitations associated with the research method. So, PCR involves the isolation of genetic material from paraffin blocks, and the obtained finding directly depends on the quality and completeness of the isolation of the material. The immunohistochemical method (IHC) for the detection of EBNA and LMP proteins (LMP proteins are cytoplasmic/membrane proteins, and EBNA proteins are nuclear ones) also has limitations which lead to a large number of false-positive results [31]. The described difficulties are also characteristic for the works that study the influence of EBV on the development of gastric cancer, though, in quite a bigger number [32, 33].

**Table 1:** Epstein–Barr virus detection studies in colorectal cancer samples

Author, year	Population	Sample size (n)	EBV status (%)	Detection method	References
Malki et al., 2020	North America	102	Detected — 20	PCR	[34]
Gupta et al., 2020	Bosnia	106	Detected — 25	PCR and IHС	[35]
Sarvari et al., 2018	Iran	210	Detected — 1.4	PCR	[36]
Al-Antary et al., 2017	Syria	102	Detected — 36	PCR and IHС	[37]
Mehrabani-Khasraghi et al., 2016	Iran	35	Not detected	PCR	[38]
Tafvizi et al., 2015	Iran	50	Detected — 38	PCR	[39]
Sole et al., 2015	Chile	37	Detected — 46	PCR	[40]
Guan et al., 2015	China	54,675 samples	Detected relationship	Microarray analysis	[41]
Fiorina et al., 2014	Italy	44	Not detected	PCR in real time and IHC	[42]
Salyakina and Tsinoremas, 2013	North America	117	Detected — 21	PCR, sequencing	[43]
Khoury et al., 2013	North America	204	Not detected	Sequencing	[44]
Delaney and Chetty, 2012	Great Britain	1	Not detected	IGC	[45]
Karpinski et al., 2011	Poland	186	Detected — 19	PCR	[46]
Chang et al., 2011	Taiwan	1	Detected	PCR	[47]
Park et al., 2010	South Korea	72	Detected — 30.6	IHC and hybridization *in situ*	[48]
Nishigami et al., 2010	Japan	1	Not detected	IGC	[49]
Militello et al., 2009	Italy	100	Detected in the material from paraffin blocks — 2.8; in the freshly frozen material — 39.3	PCR in real-time and sequencing	[50]
Song et al., 2006	China	90	Detected — 30	IHC and hybridization *in situ*	[51]
Wong et al., 2003	Scotland	26	Not detected	Hybridization *in situ*	[52]
Grinstein et al., 2002	Argentina	19	Detected — 5	Hybridization *in situ*	[53]
Kon et al., 2001	Japan	1	Detected	PCR	[54]
Kijima et al., 2001	Japan	102	Not detected	Hybridization *in situ*	[55]
Cho et al., 2001	South Korea	274	Not detected	Hybridization *in situ*	[56]
Samaha et al., 1998	North America	1	Not detected with IHC, detected with PCR	IHC and PCR	[57]
Vilor et al., 1995	Australia	1	Not detected in tumor cells, detected in intact tissue	Hybridization *in situ*	[58]
Yuen et al., 1994	China	36	Not detected	Hybridization *in situ*	[59]
Boguszakova et al., 1988	Czechoslovakia	13	Not detected	PCR	[60]
Nonoyama et al., 1974	North America	10	Detected — 10	Kinetics of DNA–DNA reassociation	[61]

Most of the modern studies have succeeded in EBV detection in CRC tissue samples by various methods, while almost all the results of twenty years ago were negative [55, 56, 59, 60]. From recent studies, a practically negative result was obtained by Sarvari et al. [36]: EBV was identified by PCR in only one sample out of 210 paraffin blocks (70 adenocarcinomas, 70 adenomas, and 70 controls). The low number of positive samples in colorectal cancer tissue is likely to be associated not with the absence of this virus in a specific tumor, but with the quality of isolation of genetic material from paraffin blocks. Thus, in the study by Tafvizi et al. [39], aimed at detecting virus DNA in CRC tissue samples with the PCR method, different results were obtained. Fragments of virus DNA were found in 38% (19/50) of colorectal cancer cases and in 50% (25/50) of cases in the group with non-malignant colon lesions (in 1/12 of adenomas and 24/38 biopsies of non-neoplastic intestinal lesions). The virus was most often found in moderately differentiated adenocarcinomas (p>0.05). It cannot be ruled out that this result is associated with a large spread of G2 tumors and not supported by specific molecular features characteristic precisely for G2 tumors. The authors found a statistically significant association between viral persistence and the stage of the disease: 42.1% (8/17) of CRC samples at stage I, 36.9% (7/27) at stage II, 15.8% (3/4) — at stage III, and 5.2% (1/2) — at stage IV were positive for EBV infection.

Similar results were obtained by Fiorina et al. [42]. When studying paraffin blocks using PCR, the virus was reported to be detected in 52% (23/44) of cases of colorectal cancer. It is noteworthy, that the edges of the resection of the patients’ surgical material were used for the virus isolation, and not the intact mucous membrane of the colon. It was found that EBV was more often observed in tumors with pronounced lymphocytic infiltration (p=0.06). *In situ* hybridization (EBER1 and -2 RNA) helped to demonstrate that the virus is located in different zones of lymphoid infiltrates, and not in tumor cells. Using IHC, the EBNA1 nuclear protein was not detected in any neoplastic cell, despite the fact that it should be expressed in both the latent and lytic phases. The IHC study did not detect the LMP1 membrane protein involved in activation either, despite the fact that the lytic cycle was confirmed by the immediate-early protein BZLF1 expressed during lytic replication (see Figure 1).

All the studies we have reviewed show virus tropism particularly to lymphoid infiltrates and are very important in understanding the molecular mechanisms of EBV in CRC, despite the fact that the authors have not noted the relationship with clinical and morphological parameters.

A study by Mehrabani-Khasraghi et al. [38] was carried out on the freshly frozen material obtained from 35 patients (15 cases of colorectal cancer and 20 cases of colon polyps): fragments of the neoplasm itself and intact mucous membrane were studied. There was no statistically significant difference between the presence of EBV and neoplasm. In some cases, virus DNA was detected in pathological areas (9/15 (60%) cases in the CRC group and 7/20 (35%) cases in the polyp group) and in the adjacent intact mucous membrane surrounding the tumor (4/15 (26.7%) cases in the CRC group and 11/20 (55%) cases in the polyp group).

The range of EBV-positive patients in various studies is quite large. So, Karpinski et al. [46], using PCR, detected the virus in 19% of cases of colorectal cancer, Sole et al. [40] — in 27% of cases, Al-Antary et al. [37] — in 36.27% of cases. These data indicate a low relationship between EBV and CRC, on the one hand, and the lack of knowledge of the prognostic role of the virus, on the other hand. Moreover, most works do not focus on the site where the virus was isolated from: from epithelial cells or the surrounding infiltrate. Given the widespread persistence of the virus and its latent form in 100% of the population, the question arises whether there is really an association between EBV and CRC.

A larger-scale study using the sequencing method was carried out by Salyakina and Tsinoremas [43]. Of 1009 cases of gastrointestinal tumors, represented by 9 different localizations, only gastric cancer and colorectal cancer showed a virus-positive result at the transcriptome level (TCGA). A statistically significant relationship between EBV and CRC was shown (p=0.02), which indicates the potential oncogenicity of this virus. However, in a similar study [44], out of 204 cases of CRC, not any EBV-positive result was identified.

In a number of works, two methods for detecting EBV were used: PCR and IHC. Thus, Gupta et al. [35] carried out a PCR study with two primers (EBNA1 and LMP1) on the material of 106 adenocarcinomas of the colon. It was found that in all cases with positive EBNA1 (15/106), LMP1 was also positive. However, 11 more cases were identified with isolated positive LMP1 (n=26), which is rather curious, since EBNA1 is expressed in latency phases I, II, and III, while LMP1 is expressed only in latency phases II and III (see Figure 1). The IHC method was used to study 63 cases of colon adenocarcinoma (antibodies to LMP1). A positive response was detected only in 7/63 cases, which is almost 2 times less compared to PCR. The result obtained by the authors makes one think about the specificity of the methods used, on the one hand, and the sensitivity of various markers, on the other hand. It should be noted that, according to IHC data, LMP1 expression was sometimes observed in the adjacent intact mucosa, and not only in the tumor itself; however, these data are not statistically confirmed.

Recent studies [62, 63] of stomach and colon cancer have focused on the identification of a new tumor suppressor gene *ARID1A* (at-rich interactive domain-containing 1A protein), which encodes a large nuclear protein involved in the regulation of a number of processes, including cell differentiation and DNA repair. *ARID1A* mutates, particularly, in CRC. A meta-analysis conducted by Kim et al. [64], which included 6 studies and 3019 patients, confirmed the presence of EBV infection and loss of ARID1A protein expression in tumors with lower differentiation and high stage.

## The role of EBV in the initiation of the epithelial-mesenchymal transition

The epithelial-mesenchymal transition (EMT), first described in the early 1980s [65], is an important process in metastasis and tumor progression in general [66]. EMT leads to losing epithelial characteristics of cells, including transitional and apical-basal polarity. EMT can reorganize its cytoskeleton and undergo multiple biochemical alterations which enable the cell to migrate and invade surrounding tissues, acquiring a mesenchymal cell phenotype. This phenotype includes increased migratory ability, invasiveness, increased resistance to apoptosis, and increased production of extracellular matrix components [67, 68]. In addition, it has been shown that the presence of inflammatory cytokines and hypoxia in the tumor microenvironment promotes the development of EMT [69].

**Figure 2:**
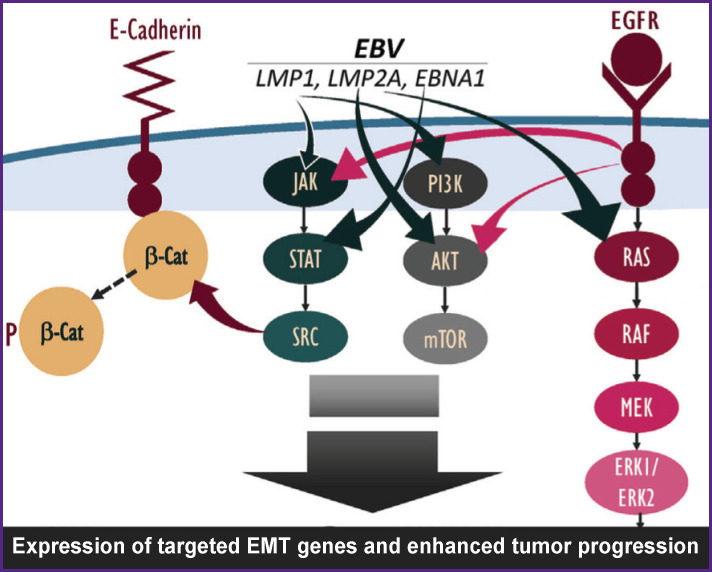
Influence of Epstein–Barr virus proteins on the development of epithelial-mesenchymal transition. Given under [8] Here: EMT — epithelial-mesenchymal transition; JAK/STAT/ SRC, PI3K/AKT/mTOR, RAS/RAF/MEK/ERK1/ERK2 — signaling pathways; EGFR — a transmembrane glycoprotein receptor

Previously, the focus of EMT studies was limited to studying the mechanisms of direct acquisition of the mesenchymal phenotype by epithelial cells. In recent decades, evidence has been accumulated that oncoviruses and the proteins encoded by them have a significant effect on metastasis, on the EMT process in particular. The EBV oncoproteins (LMP1, LMP2A, and EBNA1) have been noted to enhance the development of carcinomas through the initiation of EMT [70]. LMP1 can reduce the expression of E-cadherin by inducing a transcriptional repression complex consisting of DNA methyltransferase (DNMT-1) and histone deacetylase and induce a switch from E-cadherin to N-cadherin [71]. LMP2A is another EBV oncoprotein. It is overexpressed in the vast majority of EBV-associated nasopharyngeal carcinomas. LMP2A has been established to enhance invasive/migratory capacity and induces alterations in EMF-like cellular biomarkers [72]. Besides, LMP2A is involved in the initiation of EMT, activating the 4EBP1–eIF4E axis and, thereby, increasing the expression of metastatic tumor antigen-1. The latent EBV viral antigen, EBNA1, is a multifunctional viral protein that exhibits higher motility and migration potential, and also affects EMT markers [73]. There is evidence of the effect of miRNA on EMT and metastasis in various carcinomas, but the exact mechanisms of these processes are unknown [74]. The total effect of EBV proteins on EMT through various signaling pathways is shown in Figure 2.

He et al. [75] noted that miR-BART6-3p, which is EBV-encoded miRNA, inhibits EBV-associated migration and invasion of tumor cells of nasopharyngeal carcinoma and gastric carcinoma by reversing EMT. On the other hand, there is evidence of an increase in cadherin-6 in LMP1-positive tissues of nasopharyngeal carcinoma, which can induce EMT and promote metastasis of nasopharyngeal carcinoma [76]. Studies of miRNA in CRC are few. However, the data obtained by the sequencing method indicate a higher content of EBV miRNA in the tumor compared to the intact mucous membrane of the colon in the same patients (p≤0.05) [77].

## EBV in inflammatory bowel diseases

Since inflammatory bowel diseases are often regarded as precancerous conditions, and the nature of their occurrence does not always have a clearly described pathogen, it has been suggested that EBV influences the development of not only colon tumors but also inflammatory bowel diseases (Table 2) [78–81].

**Table 2:** Epstein–Barr virus status studies in inflammatory diseases of the colon

Author, year	Population	Disease and sample size (n)	EBV status (%)	Detection method	References
Rizzo et al., 2017	Italy	Microscopic colitis — 30 Ulcerative colitis — 30 Irritable bowel syndrome — 30	Detected — 90 Detected — 66.7 Not detected	PCR and hybridization *in situ*	[78]
Nissen et al., 2015	Holland	Ulcerative colitis — 40 Crohn’s disease — 17 Unspecified colitis — 1	Detected — 57.5 Detected — 29.4 Not detected	Hybridization *in situ*	[79]
Ryan et al., 2012	USA	Norm — 14 Crohn’s disease — 9 Ulcerative colitis — 11	Detected — 44 Detected — 55 Detected — 64	Hybridization *in situ*	[80]
Spieker and Herbst, 2000	Germany	Ulcerative colitis — 25 Crohn’s disease — 31 Collagen colitis — 8 Chronic appendicitis — 21 Chronic diverticulitis — 12 Intact mucosa — 19	Detected — 60 Detected — 80.6 Not detected Detected — 14.3 Detected — 41.7 Detected — 47.4	Hybridization *in situ*	[81]

Thus, in a study by Nissen et al. [79] performed by *in situ* hybridization, EBER cells were detected in 28 out of 58 biopsy specimens (11/16 — Crohn’s disease, 17/41 — ulcerative colitis, and 0/1 — indeterminate colitis). The relationship between the presence of EBV and atypical lymphoplasmacytic infiltrate was noted in the biopsy specimens (p<0.001). However, it remains unclear what criteria were used to consider the infiltrate as atypical (the authors describe it as a disorganized infiltrate with large B lymphocytes). There is no information about where exactly the virus was found — in the infiltrate or in the cells of the intestinal epithelium, either.

Earlier studies [80, 81] noted the presence of EBER exclusively in the lymphocytes of the lamina propria of the colonic mucosa, in lymphoid follicles, and even in the stroma of colon adenocarcinoma, but not in epithelial cells. It is noteworthy that the authors found EBV not only in samples with inflammatory disease of the colon but also in 44% of cases in the normal mucous membrane.

The study by Rizzo et al. [78] presents interest, in which the DNA of the virus was detected by PCR in 27/30 (90.0%) cases of microscopic colitis, in 20/30 (66.7%) cases of ulcerative colitis, and none of the cases of irritable bowel syndrome. Using *in situ* hybridization, EBER positive cells were identified (mainly B lymphocytes and histiocytes) in 18/30 (60.0%) cases of microscopic colitis, in 3/30 (10.0%) cases of ulcerative colitis. At the same time, the virus DNA was detected in none of the cases of irritable bowel syndrome (n=30), which is not a true inflammatory process. The authors found that EBV infection is almost always present in patients with microscopic colitis, the pathogenesis of which has not yet been established.

The research data [78–81] confirming the presence of EBV in the inflamed colonic mucosa deserve special attention, since this may indicate its influence on the development of cancer by analogy with its influence on stomach cancer.

## Conclusion

To date, there is no definite answer to the question about the direct effect of the EBV on the carcinogenesis of colorectal cancer. Until now, there is no generally accepted method for detecting the virus in tumor tissues of the colon, which leads to the incomparability of the results and the impossibility of forming a single basis. Most of the studies lack information about the location of the detected virus (epithelial cells or cells of lymphoid tissue), which is fundamentally important for assessing the stage of development of the EBV, on the one hand, and establishing its prognostic role, on the other hand. In addition, it is necessary to perform correlation analysis with clinical and morphological characteristics. Special attention should be given to the study of the role of the EBV in precancerous processes of the colon and inflammatory diseases since the virus has been identified in microscopic and ulcerative colitis.
